# Three Millennia of Vegetation, Land-Use, and Climate Change in SE Sicily

**DOI:** 10.3390/f13010102

**Published:** 2022-01-11

**Authors:** Fabrizio Michelangeli, Federico Di Rita, Alessandra Celant, Nadine Tisnérat-Laborde, Fabrizio Lirer, Donatella Magri

**Affiliations:** 1 Dipartimento di Biologia Ambientale, Sapienza University of Rome, Piazzale Aldo Moro 5, 00185 Rome, Italy; federico.dirita@uniroma1.it (F.D.R.); alessandra.celant@uniroma1.it (A.C.); donatella.magri@uniroma1.it (D.M.); 2 Laboratoire des Sciences du Climat et de l’Environnement, UMR 8212, F-91191 Gif sur Yvette, France; nadine.tisnerat@lsce.ipsl.fr; 3 Dipartimento di Scienze della Terra, Sapienza University of Rome, Piazzale Aldo Moro 5, 00185 Rome, Italy; fabrizio.lirer@uniroma1.it

**Keywords:** marine palynology, Mediterranean forest, human impact, historical ecology, Late Holocene, 2.8 ka event, Roman period, Medieval Climate Anomaly, Little Ice Age

## Abstract

This study presents the first Late Holocene marine pollen record (core ND2) from SE Sicily. It encompasses the last 3000 years and is one of the most detailed records of the south-central Mediterranean region in terms of time resolution. The combined approach of marine palynology and historical ecology, supported by independent palaeoclimate proxies, provides an integrated regional reconstruction of past vegetational dynamics in relation to rapid climatic fluctuations, historical socio-economic processes, and past land-use practices, offering new insights into the vegetation history of SE Sicily. Short-term variations of sparse tree cover in persistently open landscapes reflect rapid hydroclimatic changes and historical land-use practices. Four main phases of forest reduction are found in relation to the 2.8 ka BP event, including the Late Antique Little Ice Age, the Medieval Climate Anomaly, and the Little Ice Age, respectively. Forest recovery is recorded during the Hellenistic and Roman Republican Periods, the Early Middle Ages, and the last century. Agricultural and silvicultural practices, as well as stock-breeding activities, had a primary role in shaping the current vegetational landscape of SE Sicily.

## 1. Introduction

The Mediterranean region has been the scene of an ancient human–environment co-evolution, characterized by a complex cultural history and a considerable climatic variability that influenced the distribution and extent of forest ecosystems. From this perspective, the past few thousand years represent a period of major environmental changes, marked by unprecedented societal progress and frequent climatic fluctuations that profoundly affected the Mediterranean vegetation.

At the heart of the Mediterranean Basin, Sicily is highly sensitive to climate change due to the complex synoptic atmospheric configuration of the region [1]. This pivotal geographic location exposes forest ecosystems to the influence of the southern sub-tropical climatic regimes and the northern temperate conditions of Europe, resulting in a fragile and dynamic environment susceptible to rapid hydroclimatic variations. Furthermore, Sicily has long been a Mediterranean cultural crossroads, acting as a gateway between East and West as well as a melting pot of ancient civilizations [2]. The long history of human land use on the island left a strong imprint on ecosystems, affecting the vegetation structure and driving the evolution of rural landscapes.

Due to these features, Sicily provides a unique opportunity to study the response of forests ecosystems to past climatic events and human pressure. At the same time, its richness in natural deposits and historical documentation offers the possibility for investigating the complex human–environment interactions, cultural responses, and historical socio-economic processes linked to the changing environmental conditions of the last few millennia.

Past natural climate variability and human impact on ecosystems can be profitably studied from sedimentary archives of lakes, bogs, mires, and seafloor [3,4,5]. In particular, pollen analysis of sediment cores collected in natural deposits allows the detection of vegetation and climate trends, long-term ecological processes, and legacy effects operating over long periods of time. It provides a detailed picture of the composition, structure, and distribution of past natural vegetation and highlights the diffusion of cultivated species in relation to agricultural practices and historical land use management. Water bodies act as catchment basins for the pollen rain coming from the surrounding vegetated lands. The size of the water basins defines the dimension of the so-called pollen source areas determining the spatial scale of palaeovegetational reconstructions [6]. In this regard, terrestrial records from lacustrine deposits and small water bodies receive pollen input from a limited area surrounding the site, reflecting local vegetation changes that may not be representative of regional and supra-regional vegetation dynamics [7]. Furthermore, in continental sites, variation in perilacustrine vegetation, water level changes, local-scale factors, and human activity in proximity of the site may have a greater impact on pollen composition than the underlying climatic effects and regional trends. In contrast, marine pollen records collect pollen from vast areas and reflect regional vegetation dynamics, minimizing the small-scale processes and local factors of variability that usually affect terrestrial deposits [8,9,10]. Because of their large pollen source area, marine records are especially suited in detecting large-scale changes in relation to hydroclimatic variations and climatic events. In addition, the distance from the mainland mitigates the influence of local human-induced environmental changes and allows the tracing of land use changes at a regional scale in relation to major historical periods.

Recent palynological investigations from continental sites have provided crucial information about the Holocene vegetation and fire history of Sicily in a wide range of environments, from the coast to the highest elevations [11,12,13,14,15,16,17]. However, a comprehensive view of the regional vegetation dynamics is still missing, especially for the SE sector of the island. Furthermore, these palaeovegetational reconstructions do not provide a sufficiently detailed picture of the Late Holocene, preventing a deeper understanding of the historical vegetation dynamics in relation to cultural and climatic events.

In this study, we present the first high-resolution marine pollen record for the Late Holocene in Sicily, providing a new perspective on past vegetation dynamics at a regional scale. This new marine pollen record allows for a direct comparison with modern and past vegetation data from inland sites and correlation with the vast archaeological and documentary archives of Sicily. Our principal aim is to reconstruct the vegetation history of the last 3000 years in SE Sicily and evaluate the role of natural and anthropogenic factors in determining forest cover changes through time. In the light of the ongoing climate change, this effort helps assess the sensitivity of Mediterranean forest ecosystems and outline their response and resilience to historical land use processes and climatic variability.

Following a classical palynological approach, we frame the study area from a vegetational and climatic point of view, describe the methods used in the analysis, and interpret the pollen results in chronological succession from 3000 years ago to the present in the light of palaeoclimatic evidence from independent data and historical documentation in order to assess the role of the different natural and anthropogenic factors affecting vegetation, as well as their possible interplay.

## 2. Study Area

**Figure 1:**
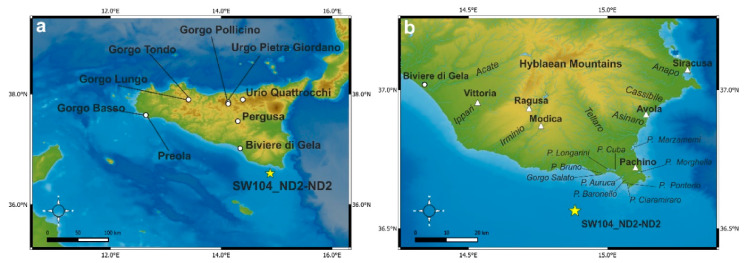
(**a**) Location map of the study site ND2 (yellow star) and pollen sites mentioned in the text (white dots): Biviere di Gela [11], Gorgo Basso [12], Gorgo Lungo, Gorgo Tondo, Gorgo Pollicino, Urgo Pietra Giordano [13], Urio Quattrocchi [14], Lago di Preola [15], Pergusa [16]. (**b**). Map of the study area with hydrographic grids of the main rivers and populated areas (white triangles). Map created using the free and Open Source QGIS 3.16.

The Hyblaean region in southeast Sicily represents the uplifted emerging portion of the Pelagian Block, corresponding to the northern offshoot of the African continental plate. The hydrography of the area consists of several rivers and seasonal streams (Acate, Ippari, Irminio, Tellaro, Cassibile, Ciane, and Anapo) that radially flow from the Hyblaean Mountains, carving natural canyons and gorges in the Oligo-Miocene outcropping formations and the underlying Mesozoic limestone (Figure 1). Near the coast, natural and semi-natural marshes, locally called “Pantani” (Pontederio, Baronello, Ciaramiraro, Auruca, Cuba, Longarini, Bruno, Gorgo Salato, Morghella, and Marzamemi), characterize a system of wet environments of significant conservation interest [18].

Regionally, different meso-climatic conditions characterize significant climatic variability from the hinterland to the coastal plains. More temperate conditions are found in the inner mountains and hills, with a mean annual temperature of 12–15 °C and mean annual precipitation of 650–850 mm, while semi-arid climatic conditions are found in the coastal areas, with a mean annual temperature of 16–19 °C and mean annual precipitation <450 mm [19,20].

The region is characterized by semi-arid open landscapes with scattered forest stands interspersed in dry Mediterranean steppes, pastures, and fallow fields. The landscape of the Hyblaean Plateau is characterized by a patchwork of dry-stone walls, rangelands, and grasslands in which relict natural forests are limited to the less anthropized areas. The distribution of forest vegetation adapts to the complex physiography of the region and reflects regional climatic variability, resulting in several bioclimatic zones [21].

Mesophilous woods of *Quercus pubescens* and *Fraxinus ornus* dominate the highest elevations of the Hyblaean Mountains, with *Laurus nobilis* in the moister zone of Monte Lauro (986 m a.s.l.). At lower elevations, the hilly landscape of the Hyblaean Plateau hosts different oak forest associations. Deciduous oak woods are mainly characterized by the *Oleo-Quercetum virgilianae*, in which *Q. pubescens* coexists with *Q. ilex* and several xerophilous woody taxa, such as *Olea europaea* subsp. *oleaster*, *Pistacia lentiscus*, and *Rhamnus alaternus*. On sub-acid volcanic soils, this vegetation is replaced by the *Mespilo-Quercetum virgilianae*, in which *Q. pubescens* and *F. ornus* are associated with *Mespilus germanica* and, to a lesser extent, with *Q. ilex*. Evergreen oak woods are mainly represented by the *Pistacio-Quercetum ilicis*, in which holm oak is found with other thermophilous taxa such as *P. lentiscus*, *Ceratonia siliqua*, and *O. europaea* subsp. *oleaster*. In the cooler slopes of the hills, the holm oak associates with *Doronicum orientale*, *Geranium robertianum*, and other mesophilous taxa within the endemic forest association of the *Doronico-Quercetum ilicis*. Mesophilous woods of *Q. ilex*, *Ostrya carpinifolia*, and *F. ornus* are found on the northern slopes of the deep river channels and in locations with high edaphic humidity. On the arenaceous and sandy substrate of the south-western sector, *Q. suber* is present with several thermo-xeric species, such as *Chamaerops humilis*, *Phillyrea angustifolia*, and *Stipa bromoides*, defining the *Stipo bromoidis-Quercetum suberis* association [22,23]. In the northern sector, sparse cork oak woodlands, with sporadic occurrences of *Q. ilex* and *F. ornus*, host two important conservation sites of *Zelkova sicula* [24,25]. From the gentle slopes of the Hyblaean Plateau to the coastal areas of the Camarino-Pachinese district, the vegetation is characterized by shrubland communities with typical Mediterranean maquis species such as *Arbutus unedo*, *C. siliqua*, *Juniperus phoenicea* subsp. *turbinata*, *O. europaea* subsp. *oleaster*, *Phillyrea angustifolia*, *P. lentiscus*, and *Calicotome infesta*, with fragmented populations of *Quercus coccifera* and widespread stands of *Pinus halepensis*. In this context, xerophytes such as *Ephedra fragilis*, *Sarcopoterium spinosum*, *Euphorbia dendroides*, and *Chamaerops humilis* become increasingly important all the way down to the coast [26,27].

## 3. Materials and Methods

### 3.1. Cores and Sampling

Two marine sediment cores (core SW104_ND2: length = 116 cm, and core ND2_2013: length = 452 cm) have been used to obtain the high-resolution composite pollen record SW104_ND2-ND2 described in this paper, hereinafter named ND2. The two records were collected in the same site from the Sicilian continental shelf, about 19 km from the coast in the Malta Channel, at a water depth of 89 m b.s.l. (36°33′52″ N, 14°52′59″ E). The cores were recovered during the NEXTDATA 2013 oceanic cruise onboard of the R/V CNR-Urania using the Kullenberg gravity corer (core ND2_2013) and the SW104 gravity corer system (core SW104_ND2), preserving the water–sediments interface. The sediment cores were wrapped and regularly sub-sampled on-site for palynological analysis. The analyzed composite core ND2 was constructed using the distribution pattern of planktonic foraminifera [28].

### 3.2. Dating

**Figure 2:**
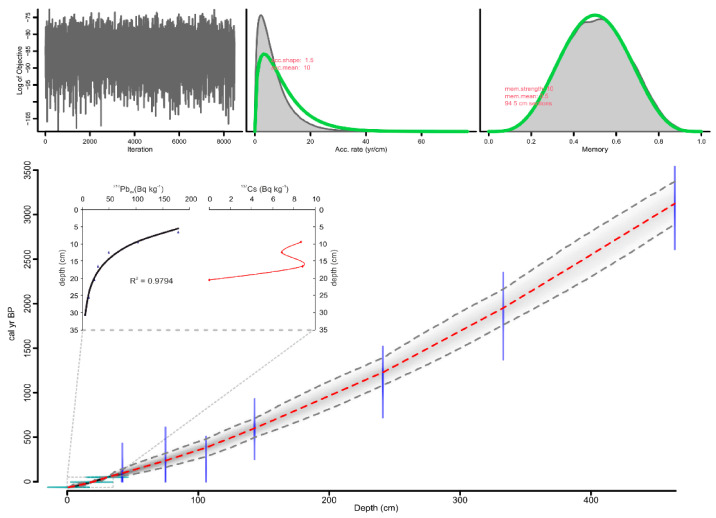
Age–depth model of the composite core ND2. Blue bars indicate the ^14^C age distribution; the greyscale of the line graph reflects the likelihood; the dotted red line follows the mean ages. Radionuclides analysis of ^210^Pb and ^137^Cs and the relative activity–depth profile is reported for the upper 30 cm [29].

The chronology of core SW104_ND2 has been previously proposed by Michelangeli et al. (2022) [29], applying the constant flux–constant sedimentation model (CF–CS) [30] to radiometric markers from short-lived radionuclides (^210^Pb and ^137^Cs) integrated with the uppermost three AMS ^14^C ages (at 42, 75, and 106 cm, respectively), as reported in Table 1. In this work, we used the short-lived radionuclides (^210^Pb and ^137^Cs) [29] and six AMS ^14^C ages (Table 1). The radiocarbon dating at 143, 241, 333, and 464 cm was performed at Laboratoire des Sciences du Climat et de l’ Environnement of Gif-sur-Yvette. Radiocarbon dates were calibrated using the calibration curve Marine20 [31], and the age-depth model was generated using a Bayesian approach with rBacon v.2.5.6 [32] (Figure 2).

**Table 1:** Radiocarbon dates.

Core	Lab. Code	Matrix	Depth (cm)	AMS^14^C Age (year BP)	AMS^14^C Error ± (years)	
SW104-ND2	Fi3219	*Turritella* spp.	42	515	55	[29]
SW104-ND2	Fi3210	shell valve	75	670	90	[29]
SW104-ND2	Fi3211	*Corbula* spp.	106	700	50	[29]
ND2_2013	GifA17420	*Globigerinoides ruber*	143	1170	55	This study
ND2_2013	GifA17572	*Globigerinoides ruber*	241	1740	60	This study
ND2_2013	GifA17571	*Globigerinoides ruber*	333	2410	70	This study
ND2_2013	GifA17417	*Globigerinoides ruber*	464	3380	65	This study

### 3.3. Pollen Analysis

**Figure 3:**
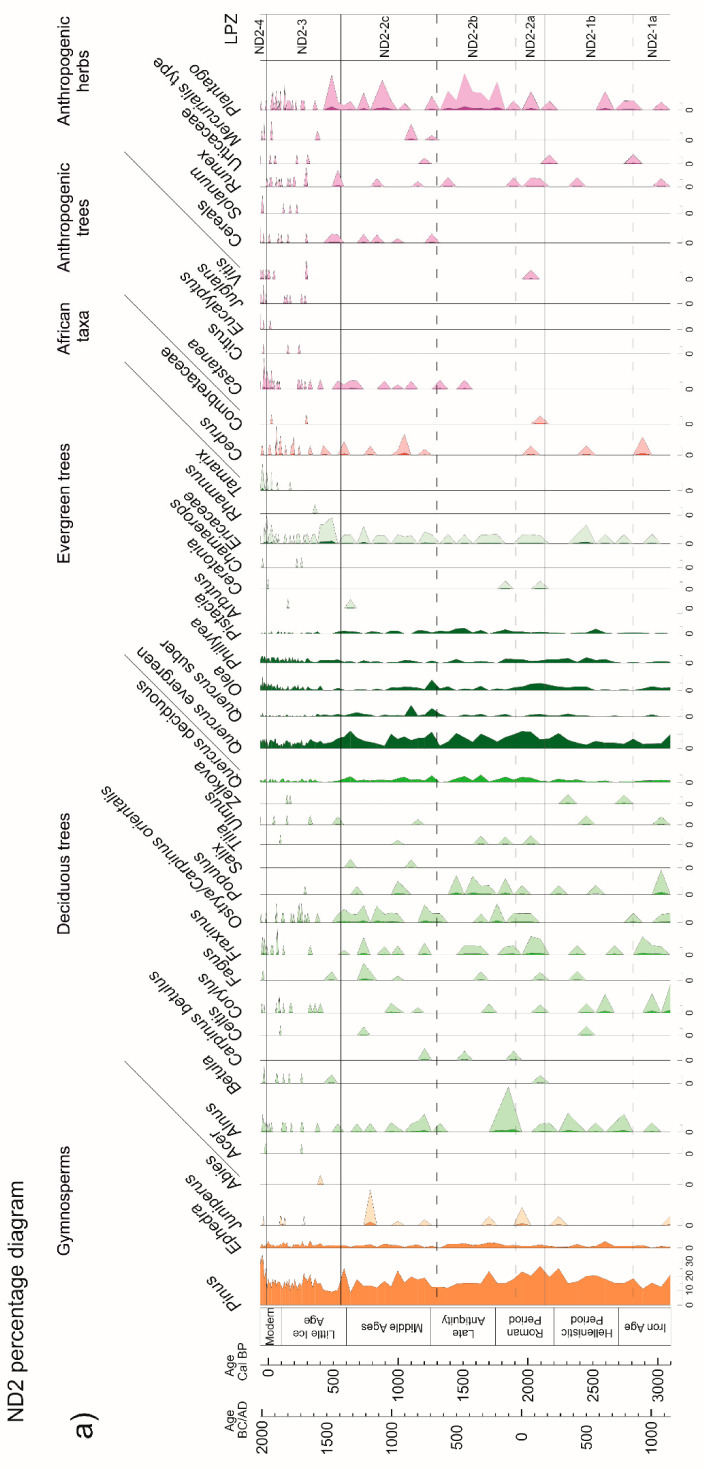

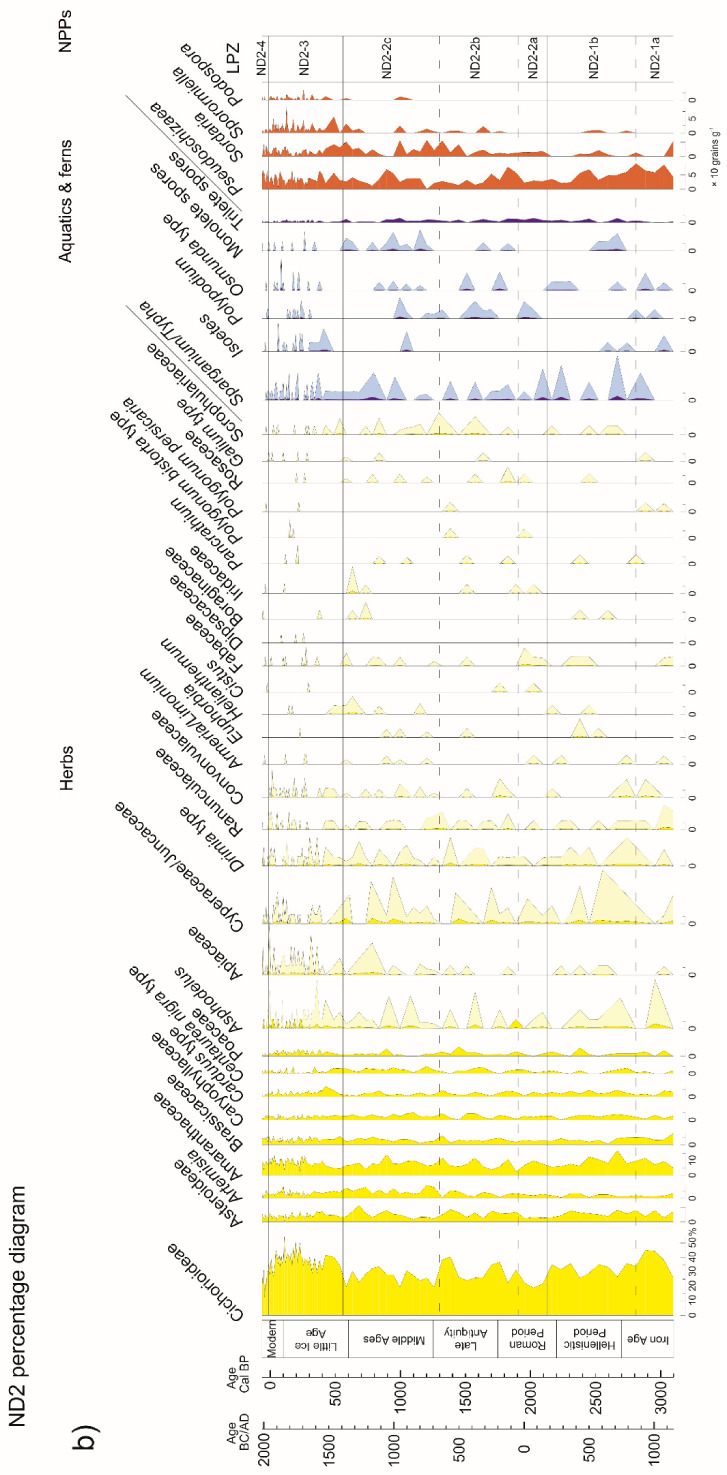
Pollen percentage diagram of selected taxa from core ND2 (×10 exaggeration in pale color). Pollen percentages of arboreal taxa (**a**). Pollen percentages of herbaceous taxa and NPP concentration (**b**). The complete pollen record is reported in Figure S1.

Chemical treatment was performed on 94 samples using HCl (37%), HF (40%), and NaOH (20%), according to the standard procedure [33]. Pollen concentration values were estimated by adding *Lycopodium* tablets to a known weight of sediment in each sample. No heavy liquids were used during sample preparation. The identification of pollen grains and non-pollen palynomorphs (NPPs) was carried out by means of light microscopy (400 to 640 magnifications) using pollen morphology atlases [34,35] and online databases [36,37,38]. Pollen counts were elaborated to obtain percentage and concentration values for each pollen type detected. The main percentage sum was based on terrestrial pollen, excluding aquatics, fern spores, and NPPs. A pollen diagram was generated representing pollen percentages in relation to the age–depth model obtained from radiocarbon chronology and short-lived radionuclides analysis on the stratigraphic sedimentary sequence (Figure 3a,b).

The pollen diagram was plotted and sub-divided into four statistically significant pollen-assemblage zones, numbered from the base upward and prefixed by the site abbreviation ND2, using the computer software Psimpoll 4.27 [39]. Constrained Cluster Analysis by Sum Squares (CONISS) was used to identify the number of statistically significant pollen assemblage zones (hereinafter “zones”), with a dissimilarity matrix of Euclidean distances, involving all the pollen taxa exceeding 2%. This agglomerative algorithm searches for the most similar stratigraphically adjacent pair of samples using the dissimilarity matrix of all pairwise combinations of samples. A sum of squares is calculated from each cluster after merging a pair of samples into one cluster, and it is recalculated as clusters are merged. The matrix is searched for two stratigraphically adjacent clusters whose merger results in the least amount of total dispersion increase. Agglomeration continues until all of the data are merged into a single cluster [40,41,42].

**Figure 4:**
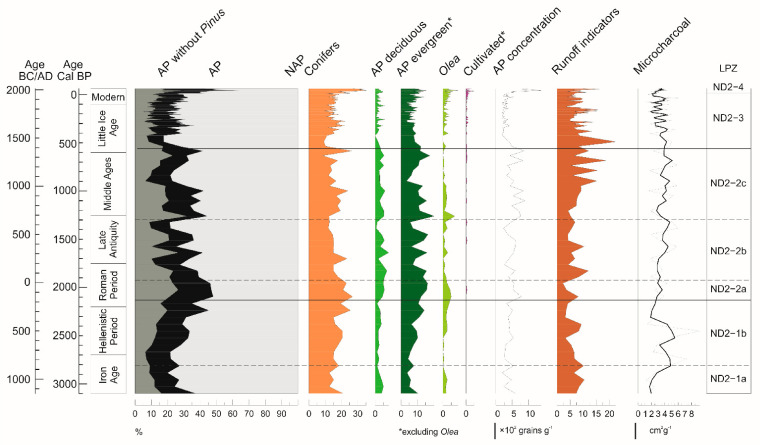
Summary pollen percentage diagram, including cumulative percentages of conifers (*Abies*, *Cedrus*, and *Pinus*), deciduous trees (mostly deciduous *Quercus*, *Alnus*, *Ostrya*/*Carpinus orientalis*, *Fraxinus*, and *Corylus*), evergreen trees and shrubs (mostly evergreen *Quercus*, *Quercus suber* type, *Phillyrea*, *Pistacia*, and Ericaceae), cultivated trees (*Castanea*, *Juglans*, *Citrus*, *Vitis*, and *Eucalyptus*), arboreal pollen %, *Olea*, runoff indicators (*Pseudoschizaea* and *Glomus* type), and microcharcoal concentration. The asterisk (*) indicates that *Olea* is excluded from the ecological group.

Additional sub-zones were visually identified based on changes in the pollen record (Figure 3a,b, Figure 4, and Figure S1).

## 4. Results

The pollen analysis was carried out on a total of 94 samples. A total of 140 pollen and spore types were identified, including 42 arboreal and 71 herbaceous taxa, 13 aquatic plants and ferns, and 14 NPPs. Moderate pollen concentration values (1271 grains/g sediment on average) reflect the distance from land and the high sedimentation rate typical of marine shelf depositional environments [8].

The pollen diagram (Figure 3a,b, Figure 4, and Figure S1) depicts a permanently open regional vegetation, with an average percentage value of arboreal pollen (AP) of 30%. The herbaceous vegetation dominates these open landscapes with several xeric elements of natural and semi-natural grasslands and pastures. Cichorioideae is the most abundant taxon, followed by Amaranthaceae, Asteroideae, *Artemisia*, Brassicaceae, and Poaceae.

Zones of uniform pollen content (pollen assemblage zones), described by hierarchical constrained cluster analysis (CONISS), are described from the base upwards to facilitate the description of the stratigraphic sequence, as follows.

*Zone ND2-1 (1150 to 200 BC)*

This is sub-divided into two vegetational sub-zones.

Sub-zone ND2-1a (1150 to 850 BC) is characterized by a decline in AP, mostly involving *Pinus* and evergreen *Quercus*. Herbs indicate an increase in Cichorioideae and Amaranthaceae and stable values of Brassicaceae and *Artemisia*. NPPs show maximum concentrations of *Pseudoschizaea* and negligible values for coprophilous fungi spores.

Sub-zone ND2-1b (850–200 BC) is initially (850–650 BC) characterized by a significant decline in *Olea* and *Quercus* deciduous. Cichorioideae dropped, while Amaranthaceae continued increasing, together with *Poaceae*, *Carduus*, and Caryophyllaceae. After 650 BC, AP increased, reaching a maximum at the end of the zone (25%), with increasing values of *Pinus, Phillyrea*, *Olea*, *Quercus* evergreen, and *Quercus* deciduous, and sporadic occurrences of *Alnus*. *Pseudoschizaea* shows a steep negative trend throughout the zone.

*Zone ND2-2 (200 BC to 1400 AD)*

Three main vegetational phases were identified.

Sub-zone ND2-2a (200 BC-50 AD) is defined by a peak in AP%, with high values of evergreen *Quercus*, *Pinus*, and *Olea*, increasing values of deciduous *Quercus* and *Pistacia*, stable values of *Phillyrea*, and a modest abundance of *Fraxinus, Ostrya, Q. suber*, *Tilia*, and Ericaceae. *Vitis* appears for the first time in the record. The general recovery of woodlands was accompanied by a marked decrease in Cichorioideae and Asteroideae pollen.

Sub-zone ND2-2b (50–650 AD) shows a steady reduction in AP of ca. 25%, culminating around 500–650 AD. *Olea* considerably decreased, along with *Phillyrea*, *Alnus*, and *Pinus*, whereas *Ephedra* increased concurrently with Poaceae and *Plantago*. Toward the end of the sub-zone, the first occurrence of *Castanea* is detected, while both *Quercus* deciduous and *Quercus* evergreen woodlands underwent a drop. This is reflected in a decrease in AP concentrations and corresponds to a general increase in herbs, especially Cichorioideae, Brassicaceae, and Amaranthaceae. NPPs show an increase in coprophilous fungi spores towards the end of the zone.

Sub-zone ND2-2c (650–1400 AD) is characterized by a general increase in AP%, although with a temporary decline in the middle of the sub-zone. After an initial rise in evergreen and deciduous *Quercus*, *Quercus suber*, *Olea*, and *Pinus*, the evergreen vegetation underwent a sudden drop (950–1150 AD), followed by a gradual recovery in the two following centuries. During this short time interval, a rise in Cichorioideae, *Plantago*, Amaranthaceae, and Cyperaceae, with appreciable values of *Artemisia* and Poaceae, is recorded. As for cultivated plants, cereals first appeared at the beginning of the sub-zone. *Castanea* persisted throughout. *Pseudoschizaea* shows oscillating values, with a peak during the AP reduction and a temporary disappearance of coprophilous spores.

*Zone ND2-3 (1400 to 1950 AD)*

This is characterized by persistently low values of AP% and decreasing AP concentration values. This zone starts with a drop of over 20% in the arboreal component, including *Pinus*, *Ostrya*, deciduous and evergreen *Quercus*, *Quercus suber*, and *Pistacia*. *Carduus* type and Cichorioideae show increasing values. Occurrences of *Plantago* and cereals are recorded. After ca. 1650 AD, there is a greater presence of cultivated plants, consisting in the first appearance of *Citrus*, *Juglans*, *Solanum,* and *Eucalyptus*, the recurrence of *Vitis,* and the presence of *Castanea*. At the same time, *Olea* shows a clear increasing trend. Long-dispersed pollen grains of *Cedrus* and Combretaceae are increasingly common, with *Cedrus* becoming more constant across the zone.

*Zone ND2-4 (1950 AD to present)*

From the 1950s, a significant increase in *Pinus* is recorded, with *Olea* and *Phillyrea* reaching their maximum. *Tamarix,* which appeared in the preceding zone, culminates here with its highest value, while Cichorioideae, *Artemisia, Carduus*, and *Centaurea nigra* type show decreasing values. Cultivated species include *Castanea*, *Citrus*, *Juglans*, *Solanum*, *Eucalyptus*, and *Vitis*, with cereals showing their highest values.

## 5. Discussion

### 5.1. The Iron Age and the 2.8 kyr Event (1150–750 BC)

At the Bronze Age–Iron Age transition (1150–750 BC), our record shows an increase in xeric herbaceous taxa, along with a decline in *Quercus* deciduous, *Corylus*, *Fraxinus*, and *Olea*. This phase represents a minimum in forest cover that has only few analogs in the last 3000 years. Various pollen records from Sicily confirm a significant forest decrease in the island during this period [13,14].

At Lake Pergusa, in the center of Sicily, an open landscape developed, characterized by rising values of Cichorioideae, Asteroideae, and Amaranthaceae. This trend was interpreted as the result of increasing dryness, causing a drop in *Olea* and an increase in xeric *Pistacia* and *Ephedra* [16].

At Gorgo Tondo, in the northern sector of Sicily, an abrupt decrease in AP of about 35% between 1150 and 750 BC led to a dramatic reduction in *Q. pubescens* and *Q. ilex*, with a temporary disappearance of *Fraxinus* and *Ulmus* and a coeval increase in Cichorioideae, Asteroideae, Amaranthaceae, and Poaceae. Cereals maintained stable low values, with intensive agriculture becoming established only during the Middle Ages; no spores of coprophilous fungi were reported [13].

At Urgo Pietra Giordano, AP was reduced by 23% between 1150 and 750 BC, mainly reflecting a drop in *Q. pubescens*, *Q. cerris*, and *Q. ilex*, while Poaceae increased. Cereals temporarily disappeared, and no coprophilous fungi spores were detected [13].

At Urio Quattrocchi in the NE sector of the island, the greatest drop in forest cover occurred, with a 66% decrease in AP between 1150 and 850 BC, followed by a gradual and steady recovery during Greek and Roman times [14]. This abrupt event affected almost all arboreal taxa and was complemented by an increase in Poaceae. Cereals show negligible values, and dung fungal spores are completely missing.

In the southern sector of Sicily, a decrease in tree cover occurred at the coastal site of Gorgo Basso, where in only one century, from 850 to 750 BC, trees underwent a terrific drop of >55%, mainly at the expenses of *Quercus* evergreen and *Olea* [12]. Forests recovered during the following century.

At Biviere di Gela, a moderate (20%) reduction in AP occurred between 1250 and 750 BC, while a steep decline in tree cover (34%) is recorded at 650 cal. BP, at the time of the foundation of the ancient city of Gela [11].

In the inland records of Sicily, the opening of forests during the first millennium BC has been associated with anthropogenic clearance, particularly with the use of fire. The transition from the Late Bronze Age (LBA) to the Early Iron Age (EIA) was characterized by rich archaeological evidence, depicting an intricate cultural context composed of different populations: the Sicels in the east, the Sicanians in the west, and the Elymians in the westernmost strip of the island [43,44]. This cultural complexity further increased with the arrival of the Phoenicians and Greeks. However, little is known of the organization and extent of these ancient societies, especially in W Sicily. The southeastern sector of the island offers rich archaeological evidence, with several rock-cut chamber tombs and necropoles documenting the demographic trends during the LBA–EIA transition [44,45]. For example, the necropolis of Cassibile, relative to a major chiefdom in the centuries preceding Greek colonization, hosted an estimated population of up to 500 people [46]. The necropolis of Montagna di Caltagirone suggests a population of 630 people during the LBA, while the site of Pantalica, with the largest number of rock-cut tombs of the island, hosted approximately 1000 people [46,47,48]. Overall, considering the size of the territory and the approximation of the estimates, these figures do not support a dense population [46].

Moreover, a review of archaeological sites in Sicily [44] reveals that the number of sites did not increase (if not decrease) from the Middle Bronze Age (MBA) to the EIA, which suggests a form of nucleation and/or demographic stability until the “Hellenization” of the island. This pattern is confirmed by recent palaeodemographic reconstructions based on summed probability distributions of calibrated radiocarbon dates (SPDs), which show a drastic demographic reduction during the LBA and EIA after a boom during the Early Bronze Age (EBA) [49].

This archaeological evidence, while bearing witness to undisputed human activity in SE Sicily, raises questions about the real impact of these indigenous populations on the natural ecosystems, particularly their ability to determine a massive opening of forests across the island between 1150 and 750 BC. On the other hand, the occurrence of an abrupt and temporary vegetational change in the Central Mediterranean during this time interval has seldom been explored in climatic terms [9,50].

Marine pollen records are particularly well-suited to detect climatic signals blurred by human activity as they minimize local-scale factors and allow for a broader perspective on past environmental changes at a regional scale [8,9,29].

In the ND2 pollen record, no unambiguous indicators of human impact at a regional scale are recorded between 1150 and 750 BC. Dung fungal spores show negligible values, and no cultivated taxa are recorded. Among the anthropogenic indicators, only moderate values of *Plantago* are found. Moreover, the decrease in *Olea*, a plant exploited in Sicily since the Neolithic [51], and certainly cultivated during the Iron Age [52,53], is unlikely to be ascribed to human activity. Indeed, a decrease in *Olea* suggests natural dynamics impacting native wild olive populations. The concomitant decline of deciduous *Quercus* supports a dry climatic phase. High frequencies of *Pseudoschizaea*, indicating runoff, point to soil erosion and downwash following deforestation, soil water repellency, and decreased soil infiltration due to prolonged droughts [54,55]. In addition, the increase in microcharcoal concentrations, which may be partly related to the Greek colonization of the island (735 BC), could have been fostered by dryness, especially in the fire-prone Mediterranean vegetation of the island.

A drought event was first observed in the marine pollen record from the Gulf of Gaeta (Central Tyrrhenian Sea), showing a 21% reduction of AP centered at 850 BC [9,56]. This vegetational change reveals a decline in *Olea* and a coeval increase in Cichorioideae, low values of secondary anthropogenic indicators, and a temporary disappearance of cultivated trees and cereals, consistently with our Sicilian record.

An increasing number of palaeoenvironmental records all around the world has documented the so-called “2.8 ka BP event” [50,57,58,59,60,61,62,63,64,65]. This climatic oscillation marked the onset of cooler and wetter conditions in Northern and Central Europe [59,66,67,68,69,70,71], with glacier advances in the Alps [72,73]. This phase also coincides with the stratigraphic event known as “Bond event 2”, recorded in the North Atlantic [74], and is concomitant to a reduction in solar activity related to the occurrence of the Homeric Grand Solar Minimum [75,76,77].

In conclusion, the forest decline observed in the ND2 core between 1150–750 BC, characterized by reduced indicators of human impact and coeval to the independent evidence for a climate event centered at 2.8 ka BP, enables a climatic interpretation of this vegetation change, which is usually interpreted in terms of human impact and Hellenistic expansion over the island.

### 5.2. The Greek and Roman Periods (750 BC–200 AD)

During the Hellenistic and Roman Republican Periods, AP% steadily rises, indicating a gradual increase in tree cover in SE Sicily, culminating in the early Roman Imperial Period.

Starting from 550 BC, the development of *Olea* is likely related to the diffusion of olive cultivation by the Greek colonists. Olive plantations in SE Sicily are documented by Thucydides in the “History of the Peloponnesian War”, in which he chronicles the retreat of the army in dry-wall-fenced olives grooves in the proximity of Syracuse during the unfortunate Athenian expedition to Sicily (Thuc. VI, 99; VII, 81). The spread of olive farming is also supported by rising pollen percentage values at Biviere di Gela after the foundation of the ancient city of Gela [11]. This vegetational signal testifies to the presence of a transformation of the landscape following the consolidation of the Greek poleis established during the previous centuries (Naxos, Megara Hyblaea, Leontini, Catane, Syracuse, Zancle, and Gela) and the foundation of second-generation colonies (Acrae, Casmenae, Kamarina, Himera, Helorus, Selinus, Heracleia Minoa, and Acragas) [78]. Interestingly, despite the spread of Greek civilization across the entire island, pollen records from northern Sicily reveal no increase in *Olea*, implying that this agricultural technique was initially developed in the south and southeast.

Agriculture was vital for the growth of these populated cities, which were dependent on cereal production from the surrounding lands [79]. Historical documentation attests to the cereal vocation of the area during the Greek establishment and later during Roman times (Diodorus Siculus, V, 69, 1–3; Homerus, Od. IX, 109–111; Cicero, Verr. II 3, 3, 179). Isotopic analysis from the city of Himera confirms that barley and wheat formed the staple foods at that time [80]. Archaeobotanical and palynological investigations in the ancient city of Kamarina reported sustainable cereal cultivation in the Chore of the city, probably in the proximity of the rivers, during the interval 600–300 BC [81]. Additional archaeological evidence for wheat cultivation in this area is related to the findings of a grain store placed in a defence tower (disrupted in 405 BC), with carbonized caryopses of *Triticum compactum* and *Hordeum tetrastichum* [82]. Despite the documentary sources and the rich archaeobotanical record, in classical times, the pollen of cereals is completely missing in the ND2 core and scarcely represented in other Sicilian sites [12,13,14,15] as it is poorly dispersed due to its shape and large size and is not easily found far from the crops [83].

Unexpectedly, between 700 and 0 BC, we found an expansion of woodland, resulting in a maximum in AP% during the first century BC, determined by the expansion of deciduous and evergreen *Quercus* and *Phillyrea*. The concurrent decrease in *Pseudoschizaea* concentrations supports a denser land cover, resulting in a reduction of runoff events.

Consistently, at Gorgo Tondo, an increase in deciduous *Quercus* characterized a marked forest recovery, as evidenced by a 30% rise in AP, peaking in the first century BC with the highest values of the entire sequence. A similar but slightly delayed vegetational dynamics is found at Urgo Pietra Giordano, where an increase in *Q. pubescens* and *Q. cerris* between 400 and 0 BC determined the highest AP% of the last 3000 years. A positive trend in woody vegetation is also found at Gorgo Lungo, with increasing values of the *Q. cerris* type. At Urio Quattrocchi, this increase in tree cover is even more pronounced (ca. 35%), encompassing the entire Greek phase until the third century AD. An increasing forest cover is also observed in central Sicily at Lake Pergusa between 650 and 50 BC [16].

At Gorgo Basso, the forest recovery (650–250 BC) was interrupted by severe anthropogenic fires, which were followed by the establishment of early successional *Pistacia* communities [14]. Similarly, at the nearby Lago di Preola, olive and oak forests were replaced by a *Pistacia* shrubland, maintaining a relatively closed canopy [15]. An analogous development of shrubby woodland is found at Biviere di Gela after the foundation of the Greek colony.

Human activity appears to have been more pronounced in the coastal sites of southern Sicily than in the northern sites, as the expansion of Greek colonists in inland mountainous areas, still inhabited by Italic populations, was limited [14].

The advent of the Romans, between the 3rd and 2nd centuries BC, is marked by rising values of *Plantago*, followed by the first occurrence of *Vitis* and high values of *Olea*.

The transition to the Imperial Period (zone ND2-2b; 50–650 AD) is characterized by a decreasing trend of AP%, lasting throughout the Late Antiquity. During this phase, *Plantago* reaches its maximum values, suggesting extensive pastureland and human disturbance on the landscape. Pollen analysis at the archaeological sites of Villa del Casale and Philosophiana revealed a significant human impact in Central Sicily, starting in the Roman Imperial Period and continuing throughout the Late Antiquity [84,85]. The vegetational landscape, characterized by agrarian systems and extensive pastures, was even more open than it is today. Unexpected low values of *Olea* match the contemporary reduced diffusion detected at many sites in southern Italy [9,11,12,13,14,15,17,86,87]. While it is improbable that *Olea* cultivation deteriorated throughout the Roman period, it is also difficult to speculate on a possible explanation, which might include unfavorable climate conditions as well as susceptibility to a pathogen analogous to modern *Xylella* [88].

### 5.3. Late Antiquity and Dark Ages (200–700 AD)

The declining trend in AP%, started during the Roman Imperial Period, continued through the Dark Ages. A temporary disappearance of *Alnus* and rising *Ephedra* values suggest increased aridity, culminating in a century-long forest cover minimum (600–700 AD) marked by a synchronous drop of both deciduous and evergreen *Quercus*. This event corresponds to the well-known short-term cooling phase of the Late Antique Little Ice Age (LALIA) [89,90,91,92].

The marine pollen record from Gaeta (Central Tyrrhenian Sea) indicates a comparable reduction in forest cover around 600 AD, marked by a steep decline in evergreen and deciduous broadleaf trees, interpreted as an arid phase in relation to a shift of the NAO index toward negative values during Bond event 1 [10]. In the Gaeta record, this event is clearly visible in natural tree populations but is partly obscured by silvicultural practices connected to *Castanea*, which was not widely spread in ancient Campania during the Roman times but became a common timber in the 5th–6th century AD. Anthropological evidence substantially supports this conclusion [93]. Chestnut appears for the first time in core ND2 around 450 AD, suggesting that chestnut cultivation spread throughout southern Italy during the post-Roman period, with a wider diffusion during the Middle Ages [94]. However, in Sicily, this spread had a limited extent compared to peninsular Italy as more restrictive hydroclimatic conditions limited its distribution to the northern sector of the island.

### 5.4. The Middle Ages and the Medieval Climate Anomaly (700–1350 AD)

After the decline in tree cover occurred during the Late Antiquity and Dark Ages, a rapid forest recovery started in the 8th century AD. During the following 250 years, woodlands and forest ecosystems may have been promoted by wetter conditions, as suggested by the general increase in both deciduous and evergreen vegetation and by the reappearance of *Alnus*.

Historically, in Sicily, this period is characterized by substantial socio-economic changes associated to the transition from Byzantine to Islamic domination. During the Middle and Late Byzantine Periods, the island underwent a considerable rarefaction of rural settlements. Nonetheless, this process was not uniform, and after the establishment of the Theme of Sicily (the province of Sicily), a marked territorial differentiation between western and eastern Sicily emerged, resulting in a prosperous eastern settlement pattern that was referred to as the capital Syracuse and was functional for both defense and agricultural productivity [95]. This process contributed to the maintenance of a sustained economy in SE Sicily even during the warfare regime associated with the recurrent Islamic incursions of the 8th and 9th centuries, as indicated by the widespread rural villages and agricultural settlements discovered by archaeological surveys [96].

This societal dynamic is reflected by a clear expansion of *Olea* in our record that started in the 8th century and persisted throughout the Byzantine and Arabic Periods (Figure 3a and Figure 4), indicating widespread olive cultivation in the Hyblaean area, which was accompanied by an intensification of cereal crops. Interestingly, the increase in *Olea* is paralleled by exceptional values of *Q. suber*, which suggests a human diffusion of this plant for cork production. A coeval exponential growth of *Olea* is found at Lago Alimini Piccolo, revealing extensive olive cultivation practices under the Byzantine administration of Apulia [86].

During the following two centuries, between 1000 and 1200 AD, forest cover underwent a new sudden and dramatic decline. This phase coincides with the so-called Medieval Climate Anomaly (MCA), a period of pronounced natural climatic variability marked by significant temperature and hydroclimatic oscillations all around the globe [97,98,99]. Since Lamb [100] first proposed an “Early Medieval Warm Epoch”, later changed to “Medieval Warm Period”, increasing evidence for this rapid climate change emerged, revealing a complex climatic scenario marked by distinct regional expressions [101]. Once again, as in the case of the forest decline centered at 2800 BP, corresponding to the Homeric minimum, the forest decrease between 1000 and 1200 AD corresponds to a variation in solar activity, related to the Oort Grand Solar Minimum [76,102], whose impact on forest ecosystems has seldom been discussed in palynological investigations [10]. In our pollen record, this change mainly affected evergreen vegetation and pine, suggesting prolonged winter droughts in the South-Central Mediterranean. Less forested landscapes and intensified soil exploitation resulted in enhanced runoff events and the leaching of the soil, as evidenced by the increasing concentrations of *Pseudoschizaea* in the sediments.

At the same time, from the Arabic to the Norman Period, the pollen record shows a slowdown of olive growing and an increase of cereal crops. Given the limited dispersion of cereal pollen [83], the detection of this pollen type in our marine record testifies to the regional relevance of cereal cultivation. Historically, this change in land-use matched the considerable socio-economic instability imposed by the wartime of the progressive Norman conquest of the island. The Norman advance conducted by Roger I began in 1061 with the conquest of Messina, followed by Maniace, Rometta, and Troina (1062). In 1063, the victory of the Battle of Cerami consolidated the Norman authority over NE Sicily [103]. In a time span of a few years, many cities such as Misilmeri (1068), Syracuse (1071), Catania (1071), Palermo (1072), Taormina (1079), Agrigento (1087), and Castrogiovanni (1087) succumbed to the Normans raids. A definitive conquest of the island followed the subjection of Butera and Noto in 1091 [104]. This pressing military campaign over the island may have contributed to destabilizing the olive cultivation fostered under the Byzantine and Arabic dominations. The presence of cereal pollen during a phase of forest reduction suggests an enhanced exploitation of the natural resources with land clearance in favor of crops. However, palaeoecological records and documental evidence on the rural and agricultural dynamics of Norman Sicily are still deficient in tracing a clear trajectory of changes, which requires a greater number of targeted investigations with higher spatial resolution.

Some information on the main features of the island is reported by the Arab geographer Al-Idrisi in the propagandistic opera commonly known as “The book of Roger”, commissioned by King Roger II around 1138. The author reported a detailed geographical description of the island, highlighting the high productivity of the lands of the main cities of the Kingdom of Sicily. However, as far as the southeastern tip of the island was concerned, apart from the woodlands in the territory of Vizzini, Buccheri, and Buscemi in the Hyblaean mountains, Al-Idrisi reported a totally desolated land for the area south of Noto (“From Noto to the eastern tip of the island which is called “Harbour of Bawalis” there is one day of walk in a totally deserted land”). This barren landscape matches the unforested territories depicted by the low AP% values of our record.

During the Norman period, forests represented a resource of primary importance in Sicily, and their exploitation was strictly controlled by severe rules regulating hunt, pasture, clearance, and timber extraction. The severity with which this legislation was applied, as well as the establishment of royal officers (*forestarii*) charged with the administration and protection of forests resources [105,106], testify to the attention of the Norman administration to the critical status of Sicilian woodlands, as also highlighted by our record.

After 1200 AD, forests re-established at the pre-MCA levels, probably in relation to less severe climatic conditions and reduced human impact on the natural ecosystems due to a demographic decline in the 13th and 14th centuries [107] and to the black death of 1347. Information about forest distribution in Sicily is extremely scarce in historical archives. However, between 1200 and 1350, the presence of a forest at Kamarina in Val di Noto (*Cammarane Foresta*) is reported [108]. Our pollen record informs us that the forest recovery was favored by an increase in *Pinus,* evergreen and deciduous *Quercus*, and *Q. suber*.

During this time, coprophilous fungi spores suggest diffused animal husbandry in the area. The coeval increase of breeding activity and oak forests recalls the historical vocation of the Hyblaean territory to the pig breeding under acorn oak groves [106]. This activity declined during the 15th century, when oak forest degraded, as indicated by historical documents [106] as well as by the pollen and NPP records.

In summary, the vegetational dynamics recorded during the Middle Ages in SE Sicily delineate a scenario of considerable complexity both in climatic fluctuations and historical events. After the forest decline of 600–700 AD, during a solar minimum [76,89], moist climatic conditions favored forest expansion through most of the Middle Ages. Between 1000 and 1200 AD, the new short-term climatic oscillation toward more arid conditions of the MCA, in conjunction with a minimum in solar activity, occurred at a time of increasing human impact and enhanced cereal cultivation, resulting in a decline of forests. The forest cover gradually recovered after the end of the MCA, peaking in 1350 AD.

### 5.5. The Little Ice Age and the Modern Period (1350 AD–Today)

After 1350, the pollen record reveals a significant change in the vegetation structure, marked by a sudden reduction in woody vegetation (>20% in one century), coinciding with the onset of the Little Ice Age (LIA) [109,110,111]. This vegetational shift culminated with the lowest AP% of the entire sequence, approximately between 1400 and 1500 AD, corresponding to the Spörer Grand Solar Minimum (1420–1530 AD) and Bond event 0 [74,112]. Almost all the arboreal species were affected by this change, with *Pinus* exhibiting a significant drop, *Quercus* evergreen showing a negative trend, and *Quercus* deciduous, *Olea, Pistacia, Ostrya*, and *Fraxinus* temporarily disappearing. The AP concentrations confirm a severe forest decline that persisted throughout the LIA until 1850, with a further minor drop around 1700, in correspondence with the Maunder Solar Minimum (1645–1715).

A similar forest decline, starting after 1300 and peaking around 1700, is found in the marine pollen record of Gaeta [9]. This forest dynamic is consistent with a change toward dryer hydroclimatic conditions in relation to a shift of the NAO toward a negative phase characterizing the entire LIA [113,114].

A huge body of archival documentation, collected by the Italian historian Trasselli, reports evidence of enhanced drought between the end of the 15th century and the first quarter of the 16th century in Sicily [115], falling within the Spörer Solar Minimum. In his pioneering work, the author carefully gathered a large number of dry historical episodes (1494, 1497, 1505, 1507, 1510, 1511, 1512, 1515, 1519, 1521 AD), emphasizing the effects of prolonged aridity in determining crop failure, livestock death, economic instability, and political disorders throughout the island.

Historical records from the city of Erice, in W Sicily, report the occurrence of rogation ceremonies associated with severe droughts, revealing the occurrence of 50 processions from 1568 to 1913. These *ad aqua petendam* (asking for rain) rituals constitute a piece of evidence of recurrent dry spells on the island, which occurred with a frequency of 2–3 years from 1568 and 1668, right in the middle of the LIA [116]. Historically, in Sicily, the LIA coincided with a period of continuous political turmoil and miserable events such as the famines of 1591, 1634, 1636, 1647, 1671, and 1793, the plague of 1624, and the earthquake of 1693 [117,118,119,120,121].

During this phase, the pollen record shows an increase in the frequency of synanthropic taxa, including *Plantago*, *Rumex*, and Urticaceae, coupled with a consistent increase in cultivated plants. Indeed, from the 17th century, cereal pollen became more frequent, *Solanum*, *Juglans*, and *Citrus* occurred for the first time, *Vitis* reappeared after a long absence since Roman times, and *Castanea* repeatedly occurred. However, the increasing anthropic pressure on the region did not correspond to any major reduction of tree cover after 1600, as evidenced by both AP percentages and concentrations. This vegetation dynamic is well confirmed by historical records for the Hyblaean territory, revealing substantially stable landscapes in terms of the proportion of woodlands and agricultural lands over the last 400 years [122]. The detection of *Zelkova* pollen, which follows sporadic previous occurrences, catches evidence of the remaining populations of *Zelkova sicula*, which are located more than 60 km away from the coring sites, and demonstrates that small populations have been restricted in hydrologic microrefugia throughout the Late Holocene [123].

High-resolution palynological investigations from core SW104_ND2 demonstrate the prominent role of historical land management policies in promoting environmental stability in relation to the exceptionally long history of feudal land use in SE Sicily [29]. Traditional agro-silvo-pastoral activities resulted in multifunctional olive agroforestry systems, integrating olive cultivation, grasslands, and breeding activities. This land-use policy promoted the persistence of dry-wall fenced surfaces characterized by sparse olive and oaks tree cover with a grazed herbaceous understory [124,125]. Even after the end of the LIA, these traditional rural landscapes did not show any significant change, except for a progressive increase in *Olea* during the 19th century in relation to less severe climatic conditions.

Significant changes in the vegetation structure occurred only during the last century with the end of the semi-feudal land use and the onset of agricultural modernization. After centuries of environmental stability, the 20th century was characterized by an abrupt increase in forest cover, mainly due to the development of *Pinus* formations. This reflects pine plantations for land reclamation during the fascist period and the *Pinus halepensis* afforestation process of the 1950s and 1960s [29,126,127]. At the same time, our pollen record shows the introduction of *Eucalyptus*, reflecting its large-scale use for forestry in the second half of the century, especially in the hinterland [128,129].

In synthesis, the 20th century represents a vegetational breakpoint marked by a complex interplay of land cover change, cultivation practices, afforestation processes, and species introduction that subverted the stability of the previous centuries, altering the landscape structure.

## 6. Conclusions

The ND2 high-resolution pollen record contributes to depicting the vegetational landscape of the Central Mediterranean during the last 3000 years. It provides detailed information on changes in forest cover, land use, stock breeding activity, cultivation, soil erosion, and the introduction of exotic plants. Our palaeovegetational reconstruction reveals the persistence of open environments with alternating phases of woodland expansion and reduction, reflecting changes in land-use practices, historical events, and climatic oscillations.

The past dynamics of tree taxa of agricultural and silvicultural relevance at a regional scale have been considered in the light of human cultural phases. The history of *Olea* is marked by three main phases of diffusion related to the Greek colonization of the island, the intensification of olive cultivation during the Byzantine–Arabic period, and the more recent spread of olive agroforestry systems. Conversely, during the pre-Hellenistic period, the Roman Imperial period, and the Norman period, *Olea* experienced marked decreases that cannot be ascribed to human activity but rather suggest a response to climatic shifts and perhaps even diseases. The record of *Castanea* reveals its post-Roman diffusion, which continued during the Middle Ages and intensified during more recent times. This result is consistent with the palynological evidence from southern Italy. However, compared to peninsular Italy, the spread of chestnut in Sicily has been limited and probably restricted to the northeastern sector of the island. Before the massive afforestation of the last century, *Pinus* appears to reflect the native coastal pine populations in Sicily, as it conforms to the natural trends in forest vegetation.

During the Late Holocene, real forest conditions are never found in SE Sicily. The sparse tree cover shows a high sensitivity to climate shifts, experiencing short-term changes that are consistent with historical climate fluctuations. Decreases in forest cover and changes in vegetation composition appear to reflect the 2.8 ka BP event, the LALIA, the MCA, and the LIA, contributing to a better understanding of the mesoscale processes of hydroclimatic changes in the Central Mediterranean and calling attention to ecosystem responses in a region highly sensitive to climate change.

## Data Availability

The pollen data from core ND2 are available from the corresponding author upon reasonable request.

## Supplementary Materials

The following supporting information can be downloaded at: https://www.mdpi.com/article/10.3390/f13010102/s1, Figure S1: Pollen percentage diagram and NPP concentrations from core ND2.
