# Supplementary material for: Three Millennia of Vegetation, Land-Use, and Climate Change in SE Sicily
Source: Forests. 2022 Jan 11;13(1):102. doi: 10.3390/f13010102 (PMC8944197; doi:10.3390/f13010102)
Supplement: Supplementary file 1 [file forests-13-00102-s001.zip › forests-1514183-supplementary.pdf]

forests

MDPI

Article

Three Millennia of Vegetation, Land-Use and Climate Change in SE Sicily

Fabrizio Michelangeli [1,*], Federico Di Rita [1], Alessandra Celant [1], Nadine Tisnérat-Laborde [2], Fabrizio Lirer[3] and Donatella Magri [1]

[1] Dipartimento di Biologia Ambientale, Sapienza University of Rome, Piazzale Aldo Moro 5, Rome, Italy
[2] Laboratoire des Sciences du Climat et de l'Environnement: Gif-sur-Yvette, Île-de-France, France
[3] Dipartimento di Scienze della Terra, Sapienza University of Rome, Piazzale Aldo Moro 5, Rome, Italy
* Corresponding author: fabrizio.michelangeli@uniroma1.it

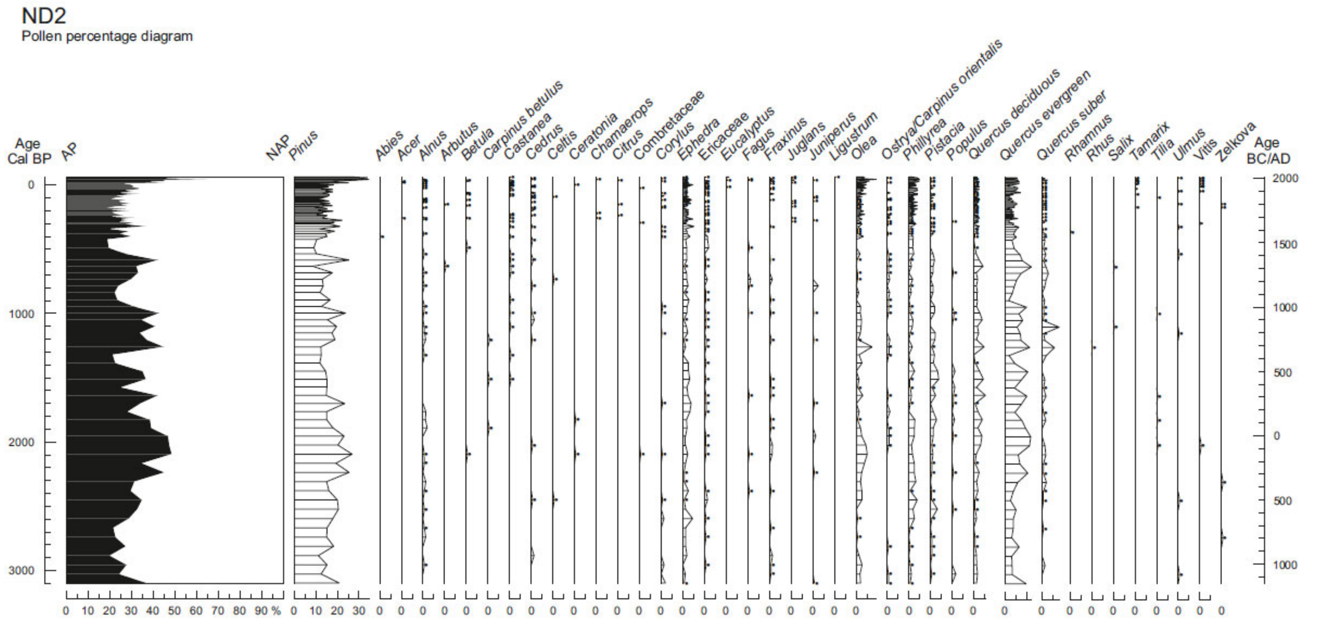

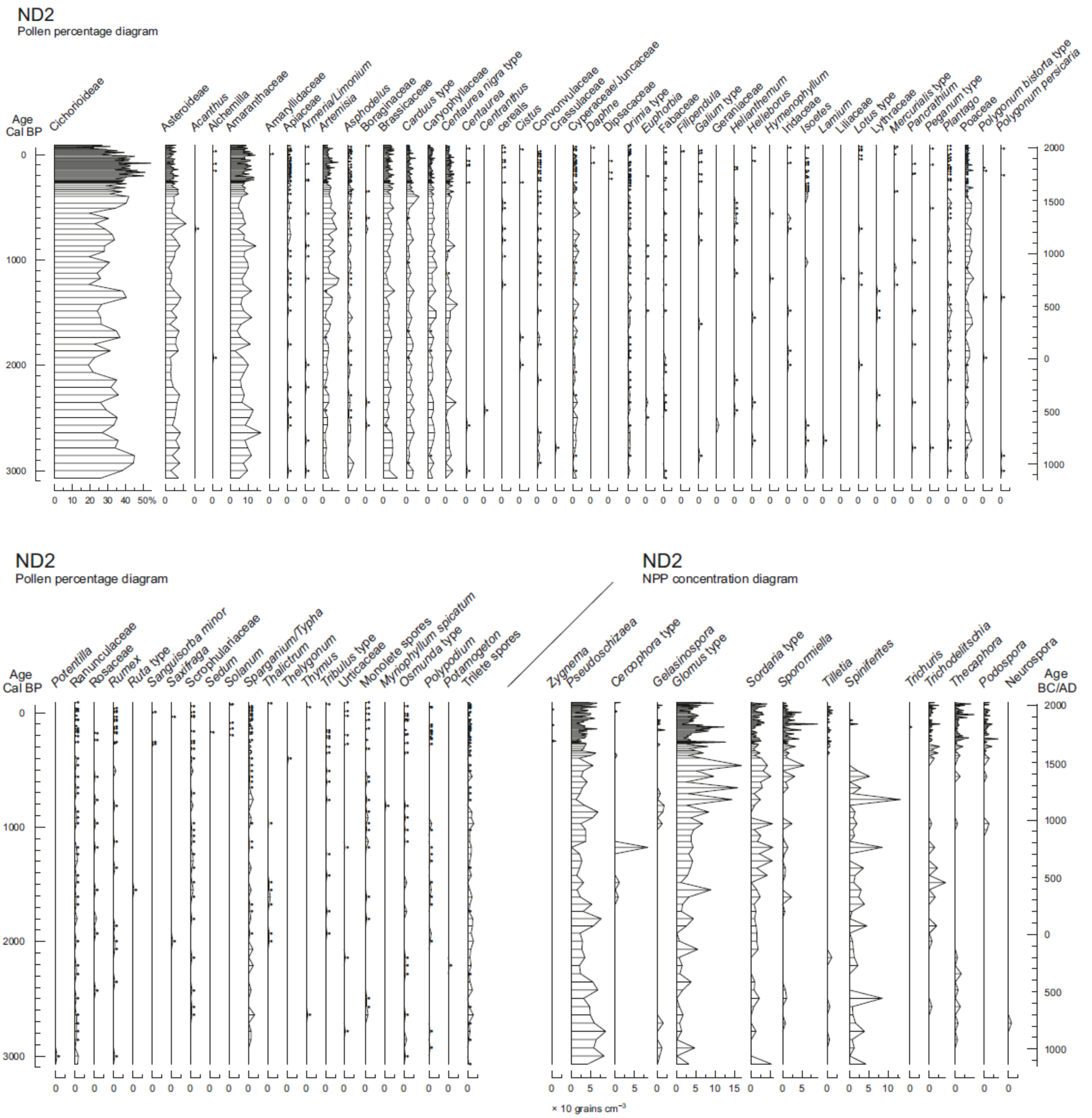

Figure S1. Pollen percentage diagram and NPP concentrations from core ND2.
